# Mental health help seeking patterns and associations among Australian same sex attracted women, trans and gender diverse people: a survey-based study

**DOI:** 10.1186/s12888-016-0916-4

**Published:** 2016-07-04

**Authors:** Ruth P. McNair, Rachel Bush

**Affiliations:** Department of General Practice, University of Melbourne, 200 Berkeley St, Carlton, 3053 Victoria Australia; School of Nursing and Midwifery, Deakin University, 221 Burwood Highway, Burwood, 3125 Australia

**Keywords:** Mental health, Help-seeking, Barriers, Enablers, Sexual orientation, Gender identity, Peer support

## Abstract

**Background:**

Same sex attracted women (SSAW) are disproportionately affected by depression and anxiety, due to experiences of sexuality and gender based discrimination. They access mental health services at higher rates than heterosexual women, however with lower levels of satisfaction. This study examined the range of professional and social help seeking by same-sex attracted women, and patterns according to sexual orientation and gender identity subgroup.

**Methods:**

Eight key stakeholders were interviewed, and a convenience sample of 1628 Australian SSAW completed an online survey in 2015. This included several scales to measure mental health, community connectedness and resilience; and measured past 12 month help seeking behaviour, enablers, barriers and preferences for mental health care. Chi-square analyses and binary logistic regression analyses examined demographic associations with mental health. Correlations between help seeking, mental and physical health, and connectedness were run.

**Results:**

A high proportion (80 %) of the total sample had perceived mental health problems over the past 12 months. Over half had depression, and over 96 % had anxiety. Trans and gender diverse participants were twice as likely as female participants to have mental health problems, and lesbians were least likely. High levels of past 12 month help seeking included 74.4 % seeing a GP, 44.3 % seeing a psychologist/counsellor, 74.7 % seeking family/friends support and 55.2 % using internet based support. Professional help was prioritised by those with higher mental health need. Trans participants were most likely to have sought professional help and participated in support groups, but least likely to have sought help from friends or family. The most common barriers to help seeking were discrimination and lack of LGBTI sensitivity of services, particularly for gender diverse, queer and pansexual participants. Enablers included mainstream community connectedness, having a trustworthy GP, and encouragement by friends.

**Conclusions:**

Mental health services need to be LGBTI inclusive and to understand the emerging diverse sexual and gender identities. Peer support is an important adjunct to professional support, however may not be fully meeting the needs of some identity sub-groups. Mental health promotion should be tailored for diverse sub-groups to build mental health literacy and resilience in the face of ongoing discrimination.

## Background

Same-sex attracted women (SSAW) are more likely than heterosexual women to experience depression, anxiety, and substance use disorders [1, 2]. In the Australian National Survey of Mental Health and Wellbeing [3], the most commonly reported past 12 month mental disorders experienced by homosexual/bisexual respondents were anxiety disorders (31.5 %), affective disorders (19.2 %), and substance use disorders (8.6 %), compared with the heterosexual sample (14.1 %, 6.0 %, and 5.0 % respectively). Specifically among women, bisexual women in the Australian Longitudinal Study on Women’s Health (ALSWH) reported the highest levels of perceived stress, depression symptoms, anxiety symptoms, and the lowest scores on the mental health index and the social support scale [4]. Transgender people also experience very high levels of mental health problems. In an Australian study of 482 trans women and 232 trans men, 43.7 % currently had clinically relevant depressive symptoms; 28.8 % had major depressive disorder; 18.3 % had a panic syndrome; and 16.9 % another anxiety disorder [5]. Moreover, 57.2 % had ever been diagnosed with depression, and 39.9 % ever diagnosed with an anxiety disorder.

Experiences of discrimination, abuse and victimisation have been consistently identified as risk factors for mental health disorders amongst these population subgroups. For example, in an Australian convenience sample of gay, lesbian, bisexual, transgender and intersex (LGBTI) people, 49.2 % of trans women and 30.9 % bisexual women had experienced at least one incident of heterosexist abuse in the previous year [6]. There was a direct relationship between having experienced abuse and higher levels of psychological distress. High levels of substance abuse are also reported amongst SSAW compared with heterosexual women [4], which is associated with mental health problems, both as a risk and causative factor. Bisexual women are even more likely than lesbians to have re-victimisation experiences and more harmful alcohol use [7]. Conversely, social support and community connectedness are protective factors for the mental health of SSAW [8, 9].

Given the higher prevalence of mental health disorders, we should expect higher utilisation of mental health services by SSAW than heterosexual women, and this is found to be the case [10, 11]. Peer support is also a common form of help seeking amongst women in general, and there is some evidence that SSAW are more likely to access self-help then heterosexual women [10]. Complementary and alternative medicine (CAM) use is also more likely amongst SSAW [12–14]. Data from the ALSWH revealed that lesbian and bisexual women were significantly more likely than heterosexual women to have used health services, however their satisfaction with these services and continuity of care were significantly lower [15]. Several barriers to effective mental health service provision for this group have been identified and categorised as external and internal barriers. External barriers are perceived inadequacies with the service such as a provider’s lack of cultural competence [16], and internal barriers include fear of mistreatment or lack of readiness to seek help [17, 18]. For example, up to 60 % of SSAW have reported negative or mixed reactions from mental health service providers towards their sexual orientation [19]. Online therapeutic services are increasingly available, but again are perceived to be inadequate. The majority of lesbian and gay people in an Australian focus group study felt that e-therapies should be more inclusive; that they should include stigma-related challenges faced by lesbians and gay men, and cater to vulnerable subpopulations [20]. Most participants believed that they were rendered invisible due to the lack of overt acknowledgement of non-heterosexuals in current online services.

There are several gaps in the literature that the current study aimed to address. Firstly, previous studies have not adequately examined help seeking behaviour and service use across the various categories of support, namely formal (professional) services including CAM, and informal services including social support and self-help. Few studies have examined patterns of help seeking among sexual minority subgroups. The majority of previous research has broadly compared heterosexuals with LGB people, and lesbian and bisexual women are often grouped together. This is problematic because sexual minority subgroups are very diverse and the issues faced by each group are not necessarily comparable [21]. Recent literature recommends the development of health promotion campaigns and messages to reduce harmful effects of substance abuse and other mental health risk factors that are tailored to specific population subgroups [22], however this is not possible until the different subgroups and their mental health service needs are identified. Therefore, the aims of the study were first to describe the mental health of the study sample and demographic associations with mental health. Second, to improve knowledge on help-seeking behaviours including types of services used by a diverse range of same-sex attracted women, based on their sexual orientation and gender identity. Third, to identify other demographic associations, barriers and enablers to help seeking. Finally, to identify priorities for targeted mental health promotion messaging for same sex attracted women.

The study hypotheses were first, that study participants would have differing levels of mental health depending on their sexual orientation and gender identity, with worse mental health amongst more marginal identity groups. This in part relates to our second hypothesis that community connectedness would be associated with better mental health, as we would expect that those with less marginal identities would be more community connected. Third, that levels of help seeking would be associated with levels of mental health need, but reduced by lack of community connectedness. Finally, we expected to identify distinct health promotion messaging that applied to same sex attracted women.

## Methods

### Study design

The study had two parts, the first involved interviews with key stakeholders and the second was a cross-sectional online survey of same sex attracted women, trans and gender diverse people. Trans people were defined as those for whom their affirmed gender differs from their assigned gender at birth. Gender diverse people were those with a non-binary gender identity. The key stakeholders were to be people working in either paid or volunteer capacities in the LGBT community in health and social support fields. They were to have experience working with same sex attracted and gender diverse people and their mental health, and have some understanding of help seeking patterns. The aim of the interviews was to understand key issues to include in the online survey, and to assist with recruitment of survey participants, particularly sub-groups to target. A further aim was to identify LGBT specific mental health promotion strategies that stakeholders had used in their work with these communities or believed would be useful. Interviews were all conducted by the first author. Stakeholders were also free to complete the online survey if they were eligible.

The Rainbow Women’s Help Seeking Study (RWHSS) survey was designed by the authors of the present study. The survey was piloted amongst LGBT researchers and people without knowledge of LGBT issues or mental health, and then some questions were made clearer and more inclusive of a wide range of SSAW, intersex and gender diverse people. The items included in the survey are described below. The eligibility criteria for survey participants were being aged over 18 years, to live in Australia, and being female (or having been socialised as women in the case of trans men and gender diverse people).

### Advertising and recruitment

Ethics approval was obtained from the University of Melbourne Human Research Ethics Committee on 20 April 2015 (ethics ID number 1543831). Key stakeholders were identified through their leadership roles within LGBT community organisations. A list was generated that purposively identified a diverse range of key stakeholders around Australia based on their geographic region, gender, paid and volunteer capacity, and target client group age. Those on the list were approached to participate in an interview. The RWHSS survey was publicised via known LGBTI websites and publications, national email and social network groups, and through mainstream websites such as *beyondblue*. Advertisements included a web address for the survey. The survey was administered online between 21 April and 17 June 2015 and hosted by www.surveymonkey.com.

### Participants

Ten key stakeholders were approached and eight agreed to be interviewed. Consent to be interviewed was obtained via email and then verbally at the beginning of the interview. Interviews were conducted in person or by phone, and audiotaped. Interviews lasted between 30 to 80 min.

A sample of 1883 people accessed the survey. A Plain Language Statement was on the homepage of the survey which provided details of the study. People were required to indicate their consent before continuing to the survey. Of the people who accessed the survey, 177 were ineligible as they did not consent to participate or did not meet the eligibility criteria. This left a total of 1706 English-speaking, LGBTI people, 18 years or older who were eligible, of whom 1628 completed all sections for analysis. The profile of the study sample is presented in Tables 1 and 2.

**Table 1:** Sample population by age bracket and identity subgroups

	Total	<30	30-50	51+	*p*
Total sample, No. (%)	1697	726 (42.8)	779 (45.9)	192 (11.3)	-
Gender Identity, No. (%)	1636				<.001
Female	1390 (85.0)	574 (41.3)	661 (47.6)	155 (11.2)	
Trans female	84 (5.1)	26 (31.0)	38 (45.2)	20 (23.8)	
Other	162 (9.9)	95 (58.6)	60 (37.0)	7 (4.3)	
Sexual Orientation, No. (%)	1624				<.001
Lesbian	848 (52.2)	242 (28.5)	476 (56.1)	130 (15.3)	
Bisexual	287 (17.7)	180 (62.7)	94 (32.8)	13 (4.5)	
Queer	234 (14.4)	104 (44.4)	113 (48.3)	17 (7.3)	
Pansexual	149 (9.2)	105 (70.5)	36 (24.2)	8 (5.4)	
Other	106 (6.5)	61 (57.5)	32 (30.2)	13 (12.3)	

Note: “Other” gender identity includes gender queer, intersex, and agender. “Other” sexual orientation includes unsure, asexual, and heterosexual (all trans or other gender identities)

**Table 2:** Demographic information with comparisons for gender identity and sexual orientation

	No.	%	Gender identity (*p*)	Sexual orientation (*p*)
Residential location, *n* = 1617			.24	.001
Inner urban	616	38.1		
Outer urban	664	41.1		
Regional centre	145	9.0		
Rural area 1^b^	142	8.8		
Rural area 2^c^	50	3.1		
Homelessness^d^, *n* = 1631			<.001^a^	.49
Never	1081	66.3		
Anytime in the past	510	31.3		
Now	40	2.5		
Indigeneity, *n* = 1630			.34^a^	.74^a^
No	1585	97.2		
Do not wish to say	7	0.4		
Aboriginal/Torres Strait Islander	38	2.3		
Ethnicity, *n* = 1614			.11^a^	.02^a^
Anglo-Australian	1225	75.9		
Anglo-European	233	14.4		
New Zealand	20	1.2		
Aboriginal	9	0.6		
Chinese, Vietnamese, Indian, Sri-Lankan	30	1.9		
Other	97	6.0		
Education, *n* = 1632			<.001^a^	<.001^a^
Still at high school	17	1.0		
School before year 12	88	5.4		
Completed high school to end of year 12	336	20.6		
Completed a trade apprenticeship or traineeship	89	5.5		
Completed a diploma	185	11.3		
Completed a university degree	567	34.7		
Completed a higher degree	345	21.1		
Other	5	0.3		
Income, *n* = 1631			<.001^a^	<.001^a^
$0-$24,999	595	36.5		
$25,000-$49,999	315	19.3		
$50,000-$74,999	299	18.3		
$75,000-$99,999	233	14.3		
$100,000-$124,999	90	5.5		
$125,000-$149,999	40	2.5		
$150,000-$174,999	29	1.8		
$175,000-$199,999	8	0.5		
$200,000 and up	22	1.3		
Relationship, *n* = 1624			<.001	<.001
No	555	34.2		
Yes, with one person	1006	61.9		
Yes, with more than one person	63	3.9		
Gender of partner, *n* = 1003				
A woman	801	79.9	.05^a^	< .001^a^
A man	170	16.9		
A trans woman	3	0.3		
A trans man	10	1.0		
Gender queer	13	1.3		
Intersex	2	0.2		
Other	4	0.4		
Have children, *n* = 1618			<.001	<.001
No	1165	72.0		
Yes	453	28.0		

Note: ^a^Monte Carlo test used due to small expected cell frequencies

^b^Population between 5000–50,000. ^c^Population less than 5,000. ^d^Includes sleeping rough/squatting, emergency accommodations, and hostels/motels etc

### Survey measures

#### Demographics

This included age, gender identity, sexual orientation, country and state or territory of residence, current or past homelessness, indigeneity, ethnicity, level of education, income, relationship status, preferred gender of partner, and number of children.

#### Community connectedness

The Connectedness to the LGBT Community Scale [23] was used to measure LGBT community connectedness. We modified it to remove references to the LGBT community in New York, and instead asked about the participant’s own LGBT community. Mainstream community connectedness was measured using a modified version of this scale by asking about their mainstream community rather than their LGBT community. Both scales contained seven items which were answered on a four-point Likert-type scale ranging from 1 (strongly disagree) to 4 (strongly agree) with high total scores indicating greater connectedness. Good reliability and validity has been found with Cronbach’s alpha ranging from .78-.81 [23]. In the current study, Cronbach’s alpha was .84 for the LGBT version and .75 for the mainstream version.

#### Perceived need for help and health

Three author developed questions asked (a) “In the past 12 months, did you think you needed help for emotional or mental health problems such as feeling sad, low, anxious or nervous?”; (b) “In the past 12 months, did you think you needed help for physical health problems?”; and (c) “Do you have a chronic (long-term) illness or disability?”. All were answered with a yes or no response.

#### Previous help seeking behaviour

The authors generated a list of health professionals, social support and self-help services, and complementary and alternative medicine (CAM) health professionals. Participants selected those they had accessed in the previous 12 months. The options for health professionals included: telephone counselling/helpline (e.g. Lifeline); general practitioner (GP); psychologist or other counsellor; psychiatrist; mental health nurse; other specialist doctor; and allied health professional (e.g. social worker, occupational therapist, and physiotherapist). Social support response options included: friends or family; religious organisations; social organisations; the internet; and mental health/emotional well-being self-help group. CAM response options included: Acupuncturist; osteopath, chiropractor, shiatsu therapist, massage therapist; and naturopath, Chinese herbalist, homeopath. “Other” options were included for each of the three questions. Degree of help seeking was measured by allocating one point to each response option that was selected and to each “other” response provided by the participant. The sum was calculated for each category to give a total score.

##### Participants were also asked

“In the past 12 months, have you accessed any LGBTI specific service?” (yes, no); “Do you have a regular GP?” (yes, no); “In the past 12 months, how often have you seen your GP?” (Never, Once/annually, 2–3 times/at least every 6 months, 4–11 times/at least every 3 months, 12 or more times/at least monthly); and “Does your regular GP know about your sexual orientation?” (I do not know, no, yes).

#### LGBTI sensitivity

Four author developed items asked, “How important is it to you for the health care services you access to be (a) LGBTI sensitive and affirming; (b) LGBTI knowledgeable; (c) LGBTI skilled (e.g. in how to assist you to disclose); (d) LGBTI-specific (i.e. established by and for the LGBTI community, such as AIDS councils or LGBTI phone counselling services).” Questions were answered on a five-point Likert-type scale ranging from 1 (not important) to 5 (very important). A Cronbach’s alpha of .81 indicated strong reliability.

#### Barriers and enablers of help seeking

The authors derived nine common barriers and eight common enablers of help seeking from the literature and participants were asked to select all that were applicable. Barriers included: concerns about confidentiality; concerns about being judged (homophobic, biphobic, transphobic attitudes); not feeling ready to seek help or advice; preferring to be self-reliant; fear of discrimination; prior experiences of discrimination; experiences of heterosexism (being assumed to be heterosexual); experiences of excessive focus on LGBTI status when it is not relevant; concerns that service providers will not be LGBTI knowledgeable and skilled; and an “other” option was included. Enablers included: having a trustworthy and sensitive GP; being out to the health care provider; having a choice of provider; knowing that a provider or service is LGBTI sensitive through personal recommendation; knowing that a provider or service is LGBTI sensitive through prior experience; knowing that a provider or service is LGBTI sensitive as it is run by a LGBTI agency or group; being able to involve your domestic partner in your health care; being encouraged to get help by a friend; and an “other” option.

#### Perceived stress

The Perceived Stress Questionnaire for Younger Women (PSQYW) [24] asked 11 questions about perceived stress over the last 12 months. Items were answered on a six-point Likert-type scale that ranged from 1 (not applicable) to 6 (extremely). Christina Lee, an author of the PSQYW, advised by personal correspondence that a score of 2 or higher could be indicative of high perceived stress. Reliability and validity has been demonstrated for this scale using a sample of 14,779 young Australian women [24]. It has also been used in both the mid aged and older cohorts of the ALSWH with good internal reliability for these age groups (http://www.alswh.org.au/for-researchers/data/data-dictionary-supplement). Cronbach’s alpha for the current sample was .72.

#### Distress

Distress over the past four weeks was measured using the Kessler Psychological Distress Scale (K10) [25]. This 10 item scale was answered on a five-point Likert-type scale ranging from 1 (none of the time) to 5 (all of the time) with a score of 30 or higher indicating clinically relevant levels of distress. Cronbach’s alpha of .94 was obtained for the current sample.

#### Depression

The 10-item Center for Epidemiologic Studies Depression Scale (CESD-10) [26] was used to measure depression. Participants answered 10 statements by indicating how often they felt or behaved that way in the past week using a four-point scale ranging from 0 (rarely or none of the time) to 3 (all of the time). A cut-off score of 10 or greater was used to indicate clinically relevant levels of depression [26]. Andresen and colleagues [26] have demonstrated a Cronbach’s alpha of .80 and the current sample obtained a Cronbach’s alpha of .89.

#### Anxiety

The Hospital Anxiety and Depression Scale Anxiety subscale (HADS-A) was used in the current study [27]. Participants ranked seven statements about their experiences of anxiety over the past week on a four-point Likert-type scale ranging from 0 (not at all) to 3 (definitely). A cut-off score of 8 or greater was used to indicate high risk anxiety [28]. The current sample obtained a Cronbach’s alpha of .77.

#### Resilience

The Brief Resilience Scale (BRS) [29] measured participants’ ability to recover from stress. Participants answered six items on a five-point Likert-type scale ranging from 1 (strongly disagree) to 5 (strongly agree). Strong reliability and validity has been found in four diverse samples with Cronbach’s alpha ranging from .80-.91 [29]. Cronbach’s alpha was .89 in the current sample.

### Data analysis

Key stakeholder interviews were audiotaped and the recordings analysed according to key themes relating to their experiences of their client group diversity amongst SSAW, help seeking attitudes and observed behaviour of SSAW, sources of assistance, and preferred LGBTI specific health promotion messages [30]. Survey data were analysed using Statistical Package for the Social Sciences (SPSS) Version 22. Simple frequencies and percentages were used to display trends in demographic characteristics and help seeking variables, and 95 % CI presented prevalence of support service access. Chi-square analyses were used to examine significant similarities and differences between age groups, sexual orientations, and gender identities on demographic characteristics and mental health indicators. Pearson’s *r* was used to examine correlations between help seeking, mental and physical health, and connectedness. Assumptions of sample size and multicollinearity were met to run binary logistic regression analyses, which were adjusted for various demographic variables. Although several outliers were detected, the decision was made to leave the cases in the analyses. A significance level of < .05 was set for all analyses.

## Results

### Sample

The eight key stakeholders included five women, one man, one trans man, and one gender queer person. Key stakeholders were not questioned about their sexual orientation. One person identified as Aboriginal. Three key stakeholders lived in Sydney, New South Wales, two were from regional centres in Queensland, one was from Victoria, one from Tasmania, and one from Western Australia, with all three of these residing in capital cities. Three of the key stakeholders were AIDS Council CEOs; one was an LGBTI mental health organisation coordinator; one was an LGBTI ageing organisation president; two were staff at a national LGBTI health organisation; and one was a LGBTI youth service coordinator.

The survey sample included 1628 people who answered a majority of the questions. The target population was any woman who was same sex attracted and people who were trans or gender diverse. People with a wide diversity of gender identities responded, a majority identifying as female, and 84 identifying as trans female (Table 1). Almost 10 % identified as other gender identities including 38 as other (gender diverse, gender queer, non-binary gender) and 10 as agender. Nine trans male participants were excluded from the analyses. Unfortunately, there were only 24 intersex identified participants, so they were analysed within the ‘other gender’ group, although we acknowledge that intersex status is not regarded as a gender identity. Lesbian, bisexual, queer and pansexual (people who are attracted to people of non-binary gender) were the most common sexual identity groups. The ‘other’ sexual orientation groups included 27 unsure, 22 asexual, 23 heterosexual (all trans or gender diverse people) and 38 listing ‘other’.

The mean age of the sample was 34.09 years with a range from 18 to 81, and there were significant differences in age according to gender identity and sexual orientation (Table 1). Lesbian identified women and trans females were more likely to be older. The majority of the bisexual, pansexual and other sexual identities were under 30, as were people with diverse gender identities. These diverse identities are emerging particularly amongst young people. By emerging, we mean they are terms and identities that have only recently been used by people over the past 5 years or so, whereas before they may have identified under the broader banners of lesbian, bisexual, or transgender.

There were several other significant demographic differences amongst the various gender identity and sexual orientation subgroups (Table 2). Female identified participants more commonly reported homelessness than gender diverse people. Lesbian and female identified respondents most frequently achieved a university degree or higher and had a higher income. Despite relatively high education levels, only 18.2 % of the sample earned more than $75,000 per annum. Lesbian and female identified participants were more likely to be in a relationship with one person than bisexual, trans female, and other gender identified participants. Lesbians were significantly more likely to report being in a relationship with a woman.

### Mental health of the survey sample

A high proportion (80 %) of the total sample perceived that they had mental health problems over the past 12 months. Perceived physical health problems were also high (73 %), and 39.5 % had a chronic illness or disability. The perception of mental health problems was correlated with high scores on the anxiety and depression scales (Table 10). As we hypothesised, there were significant differences according to gender identity and sexual orientation (Table 3). Trans females and gender diverse participants were twice as likely as female participants to be stressed (PSQYW) or distressed (K10), and more likely to be depressed (CES-D). Lesbian women were least likely of all sexual orientation groups to be stressed, distressed, depressed or anxious.

**Table 3:** Gender identity and sexual orientation differences in number of participants with clinically significant levels of self-reported symptoms of mental illness

	Perceived stress^a^		Distress^b^		Depression^c^		Anxiety^d^		Mental health problems	
	*N*	Above (%)	*N*	Above (%)	*N*	Above (%)	*N*	Above (%)	*N*	Yes (%)
Gender identity	*p* < .001		*p* < .001		*p* < .001		*p* = .10*		*p* = .01	
Female	1178	16.6	1162	27.7	1156	54.2	1152	97.4	1277	78.9
Trans female	76	30.3	76	51.3	76	67.1	76	96.1	80	85.0
Other	138	31.2	136	53.7	135	76.3	135	100.0	151	88.1
Sexual orientation	*p* < .001		*p* < .001		*p* < .001		*p* = .03*		*p* < .001	
Lesbian	739	14.2	728	23.9	728	49.0	726	96.4	798	74.8
Queer	195	25.1	194	35.1	191	60.2	191	100.0	219	85.8
Bi-sexual	242	21.9	240	37.9	238	65.1	236	98.7	262	82.8
Pan-sexual	131	32.1	128	45.3	128	73.4	128	97.7	136	91.9
Other	86	15.1	85	50.6	83	72.3	83	98.8	94	87.2

Note: *N* = total number of participants who obtained a scale score. Above % = percentage of participants above the cut-off score

*Monte Carlo test used due to small expected cell frequencies

^a^The Perceived Stress Questionnaire for Younger Women. Score range: 11–66. Cut-off score: 2 or more = high risk

^b^The Kessler Psychological Distress Scale. Score range: 10–50. Cut-off score: 30 or more = high risk

^c^The Center for Epidemiologic Studies Short Depression Scale. Score range: 0–30. Cut-off score: 10 or more = high risk

^d^Hospital Anxiety and Depression Scale, Anxiety Subscale. Score range: 0–21. Cut-off score: 8 or more = high risk

### Mental health differences according to demographic variables

Univariate analyses showed several other demographic variables that influenced mental health including age, education, income, relationship status, parenting status and homelessness. Ever experiencing homelessness had the strongest relationship of any demographics to stress (odds ratio 2.61), distress (OR 2.01) and depression (OR 1.88), and also influenced increased help seeking. These variables were added to binary logistic regression analyses for each of the four mental health variables (Tables 4, 5, 6 and 7) and after adjustment there were still several significant differences according to diverse gender identities and sexual orientations. Note that homelessness did not significantly differ among sexual orientation subgroups and therefore was only included in analyses for gender identity. Stress was influenced particularly by homelessness, lower age and income, not being in a relationship, and diverse gender identity (Table 4). Distress was strongly influenced by all demographic variables, including diverse gender and sexual identities (Table 5). Depression was affected by all demographics except parenting status, with diverse gender identity moderately influential (Table 6). Conversely, low income was the only significant demographic to influence anxiety after adjustment (Table 7). Therefore, diverse gender identities had a strong relationship to distress (OR 1.41) and moderate relationship with stress and depression. Diverse sexual orientations had a small relationship (low ORs) with distress and depression. Anxiety levels were very high across the sample, and did not significantly differ with different gender identities or sexual orientations.

**Table 4:** Binary logistic regression models for predicting high^a^ perceived stress

		Unadjusted	95 % CI			Adjusted	95 % CI	
	*p-value*	*OR*	Lower	Upper	*p-value*	*OR*	Lower	Upper
Gender identity	<.001	1.56	1.29	1.88	.02	1.27	1.03	1.56
Age	<.001	0.55	0.44	0.68	.001	0.60	0.45	0.80
Education	<.001	0.70	0.62	0.80	.30	0.93	0.80	1.07
Income	<.001	0.68	0.61	0.76	<.001	0.79	0.70	0.89
Relationship	.03	0.73	0.56	0.97	.93	0.99	0.73	1.34
Homelessness	<.001	2.78	2.19	3.54	<.001	2.61	2.01	3.38
Children	.14	0.79	0.58	1.08	.11	1.37	0.94	2.01
Sexual orientation	<.001	1.20	1.08	1.32	.41	1.05	0.94	1.17
Age	<.001	0.55	0.44	0.68	.03	0.74	0.57	0.96
Education	<.001	0.70	0.62	0.80	.06	0.87	0.76	1.01
Income	<.001	0.68	0.61	0.76	<.001	0.74	0.66	0.84
Relationship	.03	0.73	0.56	0.97	.69	0.94	0.70	1.26
Children	.14	0.79	0.58	1.08	.08	1.39	0.96	2.01

Note: ^a^Score of 2 or more on the Perceived Stress Questionnaire for Younger Women

**Table 5:** Binary logistic regression models for predicting high^a^ distress

		Unadjusted	95 % CI			Adjusted	95 % CI	
	*p-value*	*OR*	Lower	Upper	*p-value*	*OR*	Lower	Upper
Gender identity	<.001	1.82	1.53	2.17	.001	1.41	1.16	1.72
Age	<.001	0.39	0.32	0.47	<.001	0.61	0.48	0.78
Education	<.001	0.55	0.49	0.61	<.001	0.73	0.64	0.83
Income	<.001	0.60	0.54	0.66	<.001	0.78	0.70	0.87
Relationship	<.001	0.49	0.39	0.62	.03	0.75	0.58	0.98
Homelessness	<.001	2.12	1.72	2.63	<.001	2.01	1.58	2.56
Children	<.001	0.39	0.29	0.52	.04	0.69	0.49	0.98
Sexual orientation	<.001	1.36	1.25	1.49	.03	1.11	1.01	1.23
Age	<.001	0.39	0.32	0.47	.01	0.73	0.58	0.91
Education	<.001	0.55	0.49	0.61	<.001	0.70	0.62	0.80
Income	<.001	0.60	0.54	0.66	<.001	0.76	0.68	0.84
Relationship	<.001	0.49	0.39	0.62	.02	0.73	0.57	0.95
Children	<.001	0.39	0.29	0.52	.05	0.71	0.51	0.99

Note: ^a^ Score of 30 or more on the Kessler Psychological Distress Scale

**Table 6:** Binary logistic regression models for predicting high symptoms of depression

		Unadjusted	95 % CI			Adjusted	95 % CI	
	*p-value*	*OR*	Lower	Upper	*p-value*	*OR*	Lower	Upper
Gender identity	<.001	1.66	1.37	2.02	.02	1.30	1.05	1.61
Age	<.001	0.49	0.42	0.58	.004	0.73	0.59	0.91
Education	<.001	0.55	0.49	0.61	<.001	0.70	0.62	0.79
Income	<.001	0.66	0.62	0.71	<.001	0.81	0.75	0.88
Relationship	<.001	0.46	0.37	0.59	.01	0.66	0.51	0.85
Homelessness	<.001	2.07	1.66	2.58	<.001	1.88	1.47	2.40
Children	<.001	0.53	0.42	0.67	.31	0.86	0.64	1.15
Sexual orientation	<.001	1.36	1.24	1.49	.01	1.14	1.03	1.26
Age	<.001	0.49	0.42	0.58	.08	0.83	0.67	1.02
Education	<.001	0.55	0.49	0.61	<.001	0.68	0.60	0.77
Income	<.001	0.66	0.62	0.71	<.001	0.79	0.73	0.86
Relationship	<.001	0.46	0.37	0.59	.001	0.66	0.51	0.85
Children	<.001	0.53	0.42	0.67	.35	0.88	0.66	1.16

Note: ^a^ Score of 10 or more on the Center for Epidemiologic Studies Short Depression

**Table 7:** Binary logistic regression models for predicting high^a^ anxiety

		Unadjusted	95 % CI			Adjusted	95 % CI	
	*p-value*	*OR*	Lower	Upper	*p-value*	*OR*	Lower	Upper
Gender identity	.15	1.95	0.79	4.86	.17	1.96	0.74	5.18
Age	.01	0.51	0.31	0.84	.23	0.69	0.37	1.27
Education	.49	0.89	0.65	1.23	.24	1.25	0.86	1.82
Income	<.001	0.74	0.63	0.86	.001	0.73	0.61	0.88
Relationship	.79	1.10	0.54	2.26	.25	1.57	0.73	3.38
Homelessness	1.00	1.00	0.52	1.92	.82	0.92	0.46	1.83
Children	.06	0.51	0.25	1.03	.52	0.78	0.36	1.67
Sexual orientation	.05	1.41	1.00	2.00	.23	1.24	0.87	1.75
Age	.01	0.51	0.31	0.84	.29	0.72	0.39	1.33
Education	.49	0.89	0.65	1.23	.25	1.24	0.86	1.80
Income	<.001	0.74	0.63	0.86	.001	0.74	0.62	0.89
Relationship	.79	1.10	0.54	2.26	.23	1.60	0.74	3.45
Children	.06	0.51	0.25	1.03	.49	0.76	0.36	1.64

Note: ^a^Score of 8 or more on the Hospital Anxiety and Depression Scale, Anxiety Subscale

We hypothesised that community connectedness would positively correlate with mental health. We found that overall there were high levels of connection, particularly to LGBTI communities, but also to mainstream communities, relative to LGBT community sample means [23]. The levels of LGBTI and mainstream connection varied little according to gender identity and sexual orientation. We report correlations with mental health using r statistics in Table 10. Higher LGBTI connectedness was weakly correlated with higher levels of stress, but not with any of the other mental health variables. Mainstream connectedness was weakly correlated with lower levels of stress, distress, depression and anxiety.

### Help seeking for mental health issues

Overall, a high proportion of the sample had sought help over the previous 12 months, both using professional help and social support. The types of professional help seeking included 74.4 % of people seeing a GP at least once, 44.3 % seeing a psychologist/counsellor, 23.3 % seeing an allied health professional, 14 % seeing a psychiatrist, 22.2 % other specialist doctors, and 10.4 % using telephone counselling. Just 6.8 % had not accessed any professional support. Social help seeking with family and/or friends was used by 74.7 %, 55.2 % used internet support, 18 % social organisations and 10.7 % used self-help or support groups. Only 6.3 % of participants had not accessed any social support. By contrast, 44.4 % of the sample had not used any form of CAM, 33.3 % used massage therapists, 11.1 % saw a naturopath or herbalist, and 8.3 % used acupuncture. The level of help seeking varied appropriately according to mental health need (Table 8).

**Table 8:** Prevalence of help seeking by mental health indicators and perceived need for mental health support

	Perceived stress^a^		Distress^b^		Depression^c^		Anxiety^d^		Perceived MH need
	%	(95 % CI)	%	(95 % CI)	%	(95 % CI)	%	(95 % CI)	%
Professional help									
GP	85.2	(1.3-1.4)	85.4	(24.1-25.2)	85.4	(11.6-12.4)	85.3	(15.2-15.7)	84.8
Psychologist/counsellor	50.8	(1.5-1.6)	51.0	(26.6-28.0)	51.0	(13.1-14.1)	51.1	(16.0-16.7)	59.7
Allied health professional	26.6	(1.4-1.5)	26.6	(24.0-26.0)	26.7	(11.4-12.8)	26.6	(15.1-16.1)	26.3
Other specialist doctor	25.5	(1.2-1.4)	25.7	(22.9-25.0)	25.6	(10.9-12.4)	25.6	(14.7-15.6)	22.2
Psychiatrist	15.9	(1.6-1.8)	15.9	(29.7-32.2)	15.8	(15.7-17.4)	15.8	(17.0-18.3)	18.8
Telephone counselling/helpline	12.0	(1.7-1.9)	12.0	(29.0-31.9)	12.0	(14.8-17.0)	12.0	(17.4-18.8)	14.1
None	7.4	(0.9-1.2)	7.3	(20.8-24.3)	7.3	(9.2-11.5)	7.3	(14.0-15.8)	6.0
Mental health nurse	5.5	(1.8-2.1)	5.5	(32.5-36.3)	5.5	(17.3-20.2)	5.5	(18.0-20.1)	6.0
Other	3.4	(1.4-1.9)	3.4	(23.5-29.5)	3.4	(11.5-15.8)	3.4	(13.3-16.5)	3.1
Informal help									
Friends/family/partner	85.1	(1.3-1.4)	85.0	(24.0-25.1)	85.0	(11.3-12.1)	85.0	(15.2-15.7)	86.2
The internet	62.5	(1.4-1.5)	62.6	(25.5-26.7)	62.4	(12.4-13.3)	62.6	(15.8-16.4)	66.3
Social organisations	21.0	(1.5-1.7)	21.2	(25.1-27.2)	21.1	(12.0-13.5)	21.1	(15.5-16.6)	22.2
Self-help/support group	12.2	(1.6-1.9)	12.2	(28.8-31.6)	12.2	(14.4-16.4)	12.2	(16.4-17.9)	14.4
None	7.0	(0.9-1.2)	7.1	(18.7-22.8)	7.2	(8.1-11.1)	7.1	(12.6-14.5)	4.4
Religious organisations	3.4	(1.4-1.7)	3.3	(25.1-31.3)	3.4	(12.1-16.6)	3.4	(15.4-18.5)	3.5
Other	2.3	(1.3-1.7)	2.3	(20.4-25.4)	2.3	(7.8-12.8)	2.3	(12.8-15.5)	2.6
Any complimentary help	45.6	(1.3-1.4)	45.7	(22.3-23.6)	45.9	(10.3-11.4)	45.8	(14.5-15.2)	44.6

^a^The Perceived Stress Questionnaire for Younger Women. Score range: 11–66. Cut-off score: 2 or more = high risk

^b^The Kessler Psychological Distress Scale. Score range: 10–50. Cut-off score: 30 or more = high risk

^c^The Center for Epidemiologic Studies Short Depression Scale. Score range: 0–30. Cut-off score: 10 or more = high risk

^d^Hospital Anxiety and Depression Scale, Anxiety Subscale. Score range: 0–21. Cut-off score: 8 or more = high risk

^e^GP = General Practitioner

Help seeking prevalence did vary according to gender identity and sexual orientation (Table 9). Trans female participants appeared to be the highest users of services relative to other gender identities, and were also most likely to have participated in social organisations or support groups, but least likely to have sought help from friends or family. Other demographic variables were associated with higher levels of help seeking including having lower education and income levels, and living in urban areas; and younger participants were more likely to access social support. There were no differences according to relationship or parenting status.

**Table 9:** Prevalence of help seeking by gender identity and sexual orientation

	Female	Trans female	Other GI	Lesbian	Queer	Bisexual	Pan	Other SO
	%	%	%	%	%	%	%	%
Professional help								
GP	76.7	86.9	75.9	79.1	82.5	74.9	74.5	68.9
Psychologist/counsellor	43.9	57.1	57.4	41.7	54.7	46.0	58.4	47.2
Allied health professional	24.6	17.9	23.5	24.8	27.8	20.6	22.1	26.4
Other specialist doctor	22.2	40.5	19.8	23.9	27.4	20.2	18.1	21.7
Psychiatrist	11.7	44.0	22.8	12.9	17.5	13.2	16.1	22.6
Telephone counselling/helpline	9.9	13.1	17.3	8.4	13.7	12.5	16.8	12.3
None	7.5	2.4	6.2	7.8	4.7	8.4	4.0	8.5
Mental health nurse	3.7	8.3	11.7	4.5	6.8	3.5	5.4	5.7
Other	2.7	2.4	5.6	2.5	3.4	2.8	2.7	6.6
Social help								
Friends/family/partner	78.6	65.5	75.3	78.2	82.5	81.5	75.8	63.2
The internet	55.8	56.0	70.4	51.8	66.2	58.9	71.1	65.1
Social organisations	16.9	35.7	25.3	16.0	23.5	20.2	22.1	22.6
Self-help/support group	9.9	22.6	15.4	8.3	14.1	12.5	14.1	20.8
None	6.3	11.9	5.6	8.3	4.7	4.9	2.7	6.6
Religious organisations	2.4	6.0	5.6	3.2	3.0	1.0	4.0	3.8
Other	1.7	2.4	4.9	2.1	3.8	1.4	1.3	0.9
Any complimentary help	44.7	17.9	29.0	46.2	50.9	39.7	27.5	16.0

Note: *GI* Gender identity. *SO* Sexual orientation. *GP* General practitioner

Peer support groups were seen by key stakeholders as a crucial element of support for SSAW. They noted that many people turn to this type of help first before they seek professional help. However, there were several concerns about the reliance on peer support groups, including that they are often unfunded, can be difficult to find, can tend to exclude certain subgroups, and are often facilitated by people with no training in group management including conflict resolution and confidentiality. The key stakeholders identified several sub-groups that were unable to utilise peer-support within the LGBTI community due to a lack of connection (for example some older women and bisexual women) or fear of rejection including religious minority women, and women who don’t identify with any identity label. Also, preferences for family or community care over peer support were seen amongst indigenous and ethnic minority women.

General practice care was the most common source of professional help, particularly high amongst trans women (Table 9). Overall, 75.2 % of the sample had a regular GP, and 34.8 % had seen their GP at least four times over the past 12 months. Over half (56.8 %) believed that their GP knew about their sexual orientation, 18.8 % were not sure, and 24.4 % believed their GP did not know. GPs were most likely to know about sexual orientation for trans women (73.7 %) and lesbian women (71.5 %), and least likely for pansexual (30.3 %) and bisexual women (32.4 %). Having a regular GP was moderately correlated with seeking professional help (Table 10). Participants with a perceived mental health issue over the previous 12 months were less likely to have told their GP about their sexual orientation.

**Table 10:** Correlations between previous help seeking behaviour, mental and physical health indicators, perceived need for help, and community connectedness

Variables		1.	2.	3.	4.	5.	6.	7.	8.	9.	10.	11.	12.	13.	14.
1. Health professionals	*r*	1													
	*n*	1697													
2. Regular GP^a^	*r*	.21^***^	1												
	*n*	1484	1484												
3. Informal/self-help	*r*	.51^***^	.01	1											
	*n*	1697	1484	1697											
4. Comp./alt. medicine	*r*	.20^***^	.06^*^	.20^***^	1										
	*n*	1697	1484	1697	1697										
5. Perceived stress^b^	*r*	.32^***^	-.02	.26^***^	-.04	1									
	*n*	1393	1387	1393	1393	1393									
6. Distress^c^	*r*	.28^***^	-.06^*^	.21^***^	-.13^***^	.62^***^	1								
	*n*	1375	1370	1375	1375	1375	1375								
7. Depression^d^	*r*	.28^***^	-.05	.17^***^	-.10	.59^***^	.88^***^	1							
	*n*	1368	1363	1368	1368	1368	1366	1368							
8. Anxiety^e^	*r*	.22^***^	-.06^*^	.18^***^	-.09	.54^***^	.73^***^	.69^***^	1						
	*n*	1364	1359	1364	1364	1364	1363	1363	1364						
9. Emotional/mental health problems^f^	*r*	.22^***^	-	.23^***^	.01	.37^***^	.42^***^	.40^***^	.33^***^	1					
	*n*	1509	-	1509	1509	1391	1373	1366	1362	1509					
10. Physical health problems^g^	*r*	.25^***^	-	.11^***^	.14^***^	.18^***^	.11^***^	.14^***^	.10^***^	-	1				
	*n*	1502	-	1502	1502	1384	1366	1359	1355	-	1502				
11. Resilience^h^	*r*	-.28^***^	-.004	-.20^***^	.05	-.43^***^	-.55^***^	-.56^***^	-.43^***^	-.36^***^	-.11^***^	1			
	*n*	1358	1353	1358	1358	1358	1357	1356	1355	1356	1349	1358			
12. Chronic illness^i^	*r*	.36^***^	-	.15^***^	-.01	.24^***^	.28^***^	.29^***^	.22^***^	-	-	-.31^***^	1		
	*n*	1324	-	1324	1324	1324	1322	1321	1319	-	-	1322	1324		
13. LGBTI^j^	*r*	.09^**^	.01	.14^***^	.06^*^	.07^**^	.01	-.03	.04	-.02	.001	.04	.02	1	
	*n*	1560	1483	1560	1560	1393	1375	1368	1364	1508	1501	1358	1324	1560	
14. Mainstream^k^	*r*	-.05^*^	.07^**^	-.003	.05^*^	-.16^***^	-.26^***^	-.28^***^	-.18^***^	-.14^***^	-.04	.22^***^	-.09^**^	.21^***^	1
	*n*	1557	1481	1557	1557	1392	1374	1367	1363	1506	1499	1357	1323	1557	1557

Inspection of normal P-P Plots confirmed normal distributions for each mental health scale

*Note*: two-tailed *p < .05, **p < .01, ***p < .001

^a^Regular General Practitioner: 0 = no, 1 = yes. ^b^The Perceived Stress Questionnaire for Younger Women ^c^The Kessler Psychological Distress Scale. ^d^The Center for Epidemiologic Studies Short Depression Scale. ^e^Hospital Anxiety and Depression Scale, Anxiety Subscale. ^f^Emotional/Mental health problems: 0 = no, 1 = yes. ^g^Physical health problems: 0 = no, 1 = yes. ^h^Brief Resilience Scale. ^i^Chronic illness: 0 = no, 1 = yes. ^j^LGBTI: LGBTI community connectedness. ^k^Mainstream: Mainstream community connectedness

Very few participants (12.2 %, *n* = 179) had used LBGTI specific health services over the past 12 months. These services included LGBTI specific general practices, AIDS Councils, LGBTI friendly psychologists/ therapists, gender-specific services such as Gender centres and trans support groups, LGBTI support groups at University or online, and LGBTI specific youth or sexual health services. The majority of the services listed by participants were in inner urban locations, reflecting the lack of existing LGBTI specific services outside of these areas. The majority of help seeking therefore was through mainstream services.

### Non-demographic associations with help seeking

We conducted correlation analyses using r-statistics to determine relationships between help seeking and mental health, physical health, resilience, community connectedness and perceived need for help (Table 10). All four mental health variables (stress, distress, depression and anxiety) were strongly correlated with each other, and they were strongly correlated with lower resilience.

Regarding help seeking behaviour, there was a correlation between professional help seeking and social help seeking, indicating that participants generally chose to seek help in both ways rather than one replacing the other. There were medium correlations between all mental health variables and seeking professional help, and weaker relationships with seeking social support, indicating that higher mental health care need was associated more with professional help seeking. This was also reflected in medium correlations with perceived mental health problems, and less so for perceived physical health problems. Resilience was also negatively correlated particularly with professional help seeking. Perceived physical health problems was correlated with all forms of professional help-seeking.

### Barriers and enablers of help seeking in relation to sexual orientation and gender identity

We listed nine common barriers to help seeking (Table 11). Experiencing discrimination or judgement were consistently the most common barriers, followed by a lack of LGBTI sensitivity. Lack of readiness to seek help or self-reliance were common, and around one third were concerned about a lack of confidentiality. Discrimination was most problematic for gender diverse participants, and queer and pansexual people; and these same groups were also most affected by a lack of LGBTI sensitivity. Gender diverse and bisexual participants were most affected by a lack of readiness or self-reliance. Key stakeholders identified a pattern where negative experiences with mainstream care generated a lack of trust in many health professionals, and a reliance on untrained peer-support rather than professional support for some people.

**Table 11:** Barriers to help seeking for gender identities and sexual orientations

	Discrimination/judgement		Lack LGBTIQ sensitivity		Lack of readiness		Self-reliance		Confidentiality		None of these	
	No.	%	No.	%	No.	%	No.	%	No.	%	No.	%
Gender Identity												
Female, *n* = 1390	896	64.5	710	51.1	550	39.6	604	43.5	321	23.1	123	8.8
Trans female, *n* = 84	60	71.4	32	38.1	38	45.2	27	32.1	32	38.1	8	9.5
Other, *n* = 162	128	79.0	95	58.6	81	50.0	78	48.1	59	36.4	5	3.1
Sexual Orientation												
Lesbian, *n* = 848	536	63.2	421	49.6	298	35.1	347	40.9	197	23.2	93	11.0
Queer, *n* = 234	179	76.5	144	61.5	111	47.4	106	45.3	65	27.8	10	4.3
Bisexual, *n* = 287	188	65.5	143	49.8	136	47.4	150	52.3	74	25.8	20	7.0
Pansexual, *n* = 149	113	75.8	92	61.7	71	47.7	66	44.3	43	28.9	5	3.4
Other, *n* = 106	69	65.1	38	35.8	53	50.0	40	37.7	33	31.1	8	7.5

Note: Discrimination/judgement includes concerns about being judged, fear of discrimination, prior experiences of discrimination and experiences of heterosexism. Lack of LGBTI sensitivity includes experiences of excessive focus on LGBTI status when not relevant and concerns that service providers will not be LGBTI knowledgeable and skilled

Four groups of common enablers were listed in the survey (Table 12). The most commonly selected enabler was having a trustworthy GP by 62.4 % overall, followed by encouragement to get help by a friend or partner 44.6 %. Having a choice of provider was an enabler for up to one third of participants. Trans female compared with other genders and queer women compared with other sexual orientations were most likely to find LGBTI sensitivity enabling (Table 12). Encouragement to seek help by friends was most enabling for pansexual, bisexual and gender diverse participants.

**Table 12:** Enablers of help seeking for gender identities and sexual orientations

	LGBTI sensitive and knowledgeable		Awareness and acceptance by provider		Choice of provider		Encouragement by friend / Family / Partner		None of these	
	No.	%	No.	%	No.	%	No.	%	No.	%
Gender Identity										
Female, *n* = 1390	934	67.2	604	43.5	389	28.0	545	39.2	164	11.8
Trans female, *n* = 84	67	79.8	43	51.2	19	22.6	34	40.5	7	8.3
Other, *n* = 162	112	69.1	72	44.4	53	32.7	73	45.1	13	8.0
Sexual Orientation										
Lesbian, *n* = 848	595	70.2	428	50.5	243	28.7	305	36.0	94	11.1
Queer, *n* = 234	179	76.5	119	50.9	79	33.8	98	41.9	20	8.5
Bisexual, *n* = 287	177	61.7	88	30.7	80	27.9	130	45.3	40	13.9
Pansexual, *n* = 149	99	66.4	58	38.9	41	27.5	74	49.7	14	9.4
Other, *n* = 106	64	60.4	27	25.5	19	17.9	45	42.5	16	15.1

Note: LGBTI sensitive and knowledgeable includes having a trustworthy and sensitive GP, knowing that a provider or service is LGBTI sensitive through personal recommendation, knowing that a provider or service is LGBTI sensitive through prior experience, and knowing that a provider or service is LGBTI sensitive as it is run by a LGBTI agency or group. Awareness and acceptance by provider includes being out to the health care provider and being able to involve your domestic partner in your health care

Key stakeholders compared the use of mainstream health services with LGBTI-specific health services amongst SSAW. A consistent perspective was that it was particularly important for certain subgroups to have access to LGBTI inclusive mainstream mental health services. These groups included those that cannot or prefer not to access LGBTI specific services such as older women, remote or rural, cultural minorities, and indigenous women. They also identified specific services that need to be LGBTI inclusive such as those that provide primary care or deal with issues with a high degree of sensitivity. These included general practice, fertility/ gynaecology, emergency departments, women’s refuges, and domestic violence, homelessness, and mental health services.

There was also strong support for targeted mental health promotion for SSAW that highlights the specific issues affecting their mental health including discrimination, marginalisation both from mainstream and LGBTI communities, and the compounding effects of multiple identities. The key stakeholders encouraged the use of a strengths-based perspective in health promotion with three major themes: building self-esteem and normalising, encouraging help seeking including where to find LGBTI sensitive mainstream services or LGBTI specific services, and embracing diversity within LGBTI communities to overcome marginalisation.

## Discussion

The diversity of the sample in terms of sexual orientation and gender identity was broad, which we suggest reflects a social trend of diversification within the LGBTI community. No longer does the acronym LGBTI accurately reflect the range of people that are connected with this community. Other Australian researchers have recently identified specific subgroups including queer women with higher rates of anti-LGBTI discrimination than lesbians [31], and same-sex attracted men of diverse subcultural identities with more health disparities [32]. In our study, there were significant differences in the mental health and help seeking behaviours among various subgroups. Those with more significant mental health disparities tended to be the people with identities including pansexual and queer identified women, and trans and gender diverse people. This extends the existing literature on the higher rates of depression and anxiety amongst bisexual women [33, 34] and transgender women [35] to include other subgroups. Some of the contributors to bisexual differences posited in the literature include marginalisation from both mainstream and LB communities, experiences of biphobia, internalised stigma, and a lack of established bisexual community [34, 36, 37]. Distress amongst trans people is related to being victimised, lack of family support and lack of connection with LGBT peers [35]. Marginalisation even within LGBTI communities is also likely to be influencing the mental health of the diverse subgroups that we have identified. We had expected that LGBTI community connection would correlate with better levels of mental health as seen in other work [38, 39], however this was not the case, and indeed higher perceived stress was slightly correlated. It is possible that our results were influenced by issues of marginalisation amongst various subgroups, so that connection was being sought, but expectations of support were not being met due to identity policing, rejection or lack of organised and specific peer groups.

A further subgroup of note in our study were the people who had ever experienced homelessness (33.8 % of the sample). LGB people are over-represented in Australian national homelessness data, with 13.4 % heterosexuals ever having been homeless compared with 20.8 % bisexual people and 33.7 % lesbian/gay people [40]. Higher rates and subsequent impacts of homelessness for LGBT people is an emerging issue [41, 42] and should always be considered when examining the influences on mental health and help seeking disparities for LGBTI people.

Levels of depression, anxiety and stress in our study were much higher than those in an Australian population-based sample from the Australian Longitudinal Study of Women’s Health (ALSWH), despite similar demographic findings including income and education levels [4]. For example, the mean perceived stress score in that study was 0.88 for heterosexual women, 1.04 for lesbians, and 1.34 for bisexual women (*p* < .01); compared with scores in our study of 3.13 for lesbians and 3.36 for bisexuals. Similarly, proportions of women scoring in the depressed range on CES-D in the ALSWH varied with heterosexuals at 24.5 %, lesbians 28.6 % and bisexuals 44.4 %; compared with lesbians at 49.0 % and bisexuals at 65.1 % in our study. Therefore, we suggest that our study attracted people with much higher levels of mental health problems than expected for Australian same-sex attracted women generally, probably as our study was overtly focused on help seeking for mental health.

The levels of health service use for mental health problems over the previous 12 months were higher in our sample than those in the 2007 National Survey of Mental Health and Wellbeing. In our study, professional service use amongst people with depression included 85.4 % seeing a GP, 51 % seeing psychologist and 15.8 % seeing a psychiatrist; compared with 58.6 % seeing any service in the national survey [43]. The use of services for those with anxiety in our study were 85.3 % seeing a GP, 51.1 % seeing a psychologist and 15.8 % seeing a psychiatrist, compared with 37.8 % seeing any service in the national survey. Even for those with severe mental health problems in the national survey, the rates of use in the past 12 months are much lower than in our survey for GP use 49.7 % (CI 39.6-59.8), psychologist 23.1 % (CI 16.1-30.1), but higher than our survey for psychiatrist use 19.4 % (CI 12.0-26.8) [44]. So, it seems that professional health service use for equivalent levels of depression and anxiety in our study was higher than in the national study, indicating a willingness amongst people of diverse sexual and gender identities to seek help. This was also found in a UK population-based study, with higher usage of general practice and community support amongst the non-heterosexual group compared with heterosexuals [1]. By contrast, the level of complimentary help seeking did not vary with the type or severity of mental health need. We consider that this suggests participants may have used this support for health maintenance rather than mental illness management.

Social support help seeking rates were very high across the range of participants in our study, and were correlated with all four mental health indicators, and perceived mental health problems. We consider that peer support is a crucial element of mental health care for these population groups, particularly as it is likely to contribute to high levels of encouragement by peers to seek help. In our sample, this encouragement was perceived to be an enabler of help seeking, particularly for the more potentially marginalised populations, such as gender diverse, trans, pansexual and bisexual identified people. They were also most likely to identify both discrimination/fear of judgement and a lack of readiness as barriers to professional help seeking. Fear of discrimination related to diverse sexual orientations and gender identities is found to be a major barrier to seeking professional care, particularly amongst young and gender diverse people [18, 45]. It is also a pattern for older LGB people, with evidence that they are more likely to rely on partners than professional services [46]. There is evidence that formalised peer support groups improves mental health for lesbian and bisexual women [47]. It also provides people with information about LGBTI sensitive services to attend, and encourages professional support [48]. The internet was used by the majority of our participants across the age spectrum for social support and this should be recognised as an increasingly legitimate source of help [18]. Further, broader social interventions to reduce discrimination and improve mental health should be encouraged including mainstream community and family support.

There are implications for mainstream health services arising from our study, in particular that they need to be LGBTI sensitive. First, few participants accessed LGBTI specific services, relying instead on mainstream services; second key stakeholders identified the need for mainstream services to be more LGBTI inclusive, and finally over two thirds of the participants identified access to such services as an enabler of help seeking. Yet there is evidence that very few health services actually offer such care. An Irish study found that 64 % participants experienced a lack of LGBT knowledge amongst mental health professionals [49]. Further, LGBTI sensitivity means understanding the diversity within this population group, rather than treating all LGBTI people in the same way. For example, “concordance” of sexual identity and behaviour is found to be associated with higher levels of health screening than for women with discordant identities [50]. People with ‘discordant’, perhaps better phrased as diverse or fluid, expressions of sexual identity, sexual attraction and gender identity tend to have more difficulty being understood and their identities can be de-legitimised. This might explain why so many of the trans (86 %) and lesbian (79 %) women had disclosed to their regular GP in our study, compared with so few bisexual (32 %) and pansexual (30 %) women. Further, the gender diverse participants, queer and pansexual people were most likely to identify discrimination in services as a key barrier. These are all identities that are more marginal, and providers are likely to have low levels of knowledge about their specific health needs. Reluctance to disclose may also relate to personal or friends’ experiences of lack of sensitivity in the face of fluid and emerging identities. Yet, given that these very experiences in their wider lives are contributing to their mental health needs, LGBTI sensitive care becomes a critical need. A Sydney-based study of LBQ women found that the queer-identified women had experienced the highest levels of sexuality-based discrimination, had the highest levels of illicit drug use, and were most likely to seek professional support [31]. The authors concluded that “meaningful engagement with contemporary sexual identities and their local social and cultural significance is essential for the development of appropriate and effective targeted public health interventions.” Recent development of online mental health services that are tailored to LGBTI people may be an additional source of LGBTI sensitive care [51].

The study limitations included, first, that this was a convenience sample, so cannot be generalised to all SSAW. Particular groups that are not well represented include those that are less connected with LGBTI communities, and people with intersex status. Second, the cross-sectional study design was useful to provide descriptive analyses, however does not allow delineation of causation or directions of association. For example, the question of whether LGBT community connectedness increases mental health vulnerability due to feelings of marginalisation within the community cannot be answered. Third, the correlations are also limited to linear associations and cannot assist with more complex associations such as the relative influence of both stress, marginality and homelessness on depression. Finally, the study attracted a disproportionate number of people with mental health problems, so does not well represent help seeking behaviours for mental health maintenance by SSAW with good mental health. However, the convenience sampling also leant strength to the study, through providing large numbers of people and allowing disaggregation and comparison of several large sub-groups.

## Conclusions

The level of help seeking was appropriately high, given the high degree of mental health need in the sample. Help seeking was well balanced between professional services, particularly general practice and counselling; and peer-based social support, particularly from friends and via the internet. However, there were significant barriers to help seeking including a lack of LGBTI sensitivity and knowledge amongst mainstream services, and internal barriers of self-reliance and lack of readiness. This indicates that while help seeking was high, the value of the help received may have been inadequate. Professional help can lack the necessary understanding of sexuality and gender based discrimination, and social support can fail to embrace the diverse range of SSAW needing help. Future in-depth qualitative research would help to identify best practice approaches with this population group. In addition to the need for primary care and mental health services to build their sensitivity, it would also be useful to develop tailored mental health promotion messaging for each of the main sub-groups identified in the study. This should be co-designed with SSAW consumers to create mental health literacy and engagement with both social and professional help seeking. A particular aim of such messaging would be to build resilience in the face of persistent threats to their mental health.

## Abbreviations

ALSWH, Australian longitudinal study of women’s health; CAM, complementary and alternative medicine; CESD-10, 10-item center for epidemiologic studies depression scale; HADS-A, Hospital Anxiety and Depression Scale Anxiety subscale; K10, Kessler psychological distress scale; LGBTI, lesbian, gay, bisexual, transgender and intersex; PSQYW, perceived stress questionnaire for younger women; SSAW, same-sex attracted women
